# Synthesis and properties of porous polymers synthesized by Michael addition reactions of multi-functional acrylate, diamine, and dithiol compounds†

**DOI:** 10.1039/c9ra09684a

**Published:** 2019-12-23

**Authors:** Naofumi Naga, Shun Fujioka, Daisuke Inose, Kumkum Ahmed, Hassan Nageh, Tamaki Nakano

**Affiliations:** Department of Applied Chemistry, College of Engineering, Shibaura Institute of Technology 3-7-5 Toyosu, Koto-ku Tokyo 135-8548 Japan; Graduate School of Engineering and Science, Shibaura Institute of Technology 3-7-5 Toyosu, Koto-ku Tokyo 135-8548 Japan; SIT Research Laboratpries, Shibaura Institute of Technology 3-7-5 Toyosu, Koto-ku Tokyo 135-8548 Japan; Institute for Catalysis and Graduate School of Chemical Sciences and Engineering, Hokkaido University N 21, W 10, Kita-ku Sapporo 001-0021 Japan; Integrated Research Consortium on Chemical Sciences (IRCCA), Institute for Catalysis, Hokkaido University N 21, W 10, Kita-ku Sapporo 001-0021 Japan

## Abstract

Porous polymers have been synthesized by Michael addition reactions of multi-functional acrylate and diamine or dithiol compounds. Aza-Michael addition reaction of multi-functional acrylate, trimethylolpropane propoxylate triacrylate (TPT) and hexamethylene diamine (HDA) in dimethyl sulfoxide (DMSO) successfully yielded the porous polymer. The porous structure was characterized by connected globules or co-continuous structure, and could be controlled by the reaction conditions. Mechanical properties of the porous polymers were investigated by compression test. The porous polymers with co-continuous structure showed higher Young's modulus than those with connected globules. The porous polymer absorbed some organic solvents, especially CHCl_3_. The porous polymer as prepared in DMSO state showed coloring induced by Christiansen filter effect depending on the reaction time and observation temperature. The thio-Michael addition reaction of TPT and 1,6-hexanedithiol (HDT) in DMSO using different base catalysts also yielded the porous polymer. The porous structure could be controlled by the catalysts amount when the reaction was initiated by a photo-base generator as the base catalyst. The present reaction systems make it possible to synthesize the porous polymers with simple process without phase separator.

## Abstract 2

Porous polymers have been synthesized by Michael addition reactions of multi-functional acrylate and diamine or dithiol compounds.

## Graphical abstract

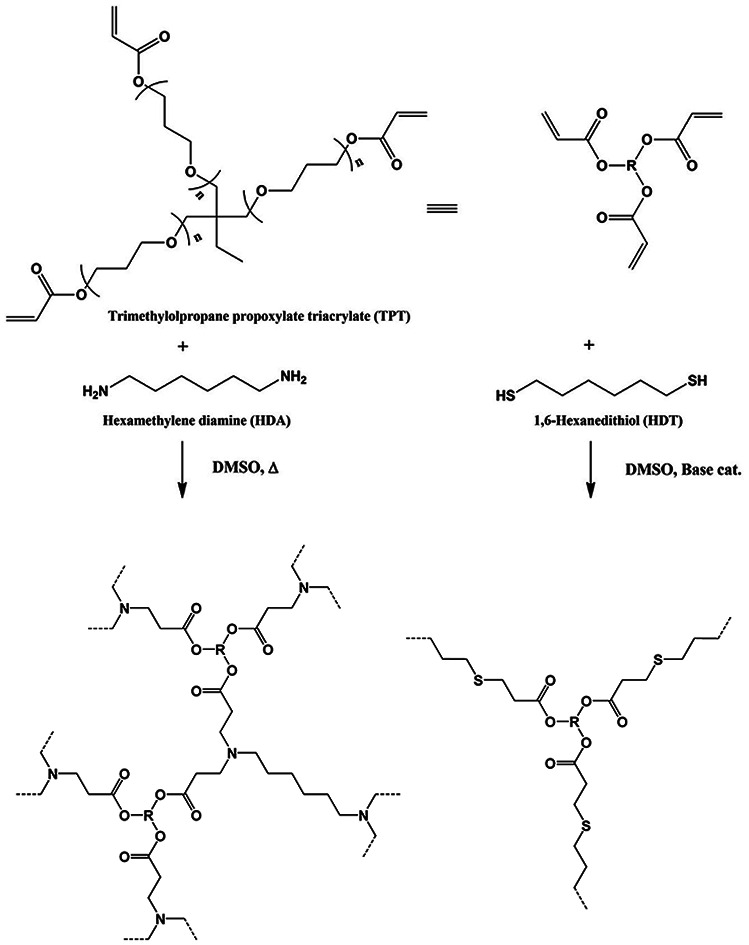

## Introduction

Porous organic polymer materials with a monolithic, co-continuous structure of polymer and space, are applied in separation filters, columns for chromatography, supports for catalysts, electrochemical cells and so on.^1^ Monolithic organic polymers have been prepared *via* phase separation process in the (reaction) system induced by temperature change of polymer solutions or polymerization of vinyl-type monomers. Several types of porous polymers have been synthesized from some click-type polymerization reactions, such as addition reaction of multifunctional epoxy compounds with amine^2^ or thiol^3^ compounds accompanied by ring opening of the epoxy groups, thiol-ene/yene,^4^ thiol-(meth)acrylate reactions,^5^ in the presence of pore-generator. In the case of the polymerization induced phase separation, correlation between the rates of polymerization and phase separation should be important to form and control the monolithic structure of the network polymers. Mixed solvent system and/or pore-generator, were usually used to obtain the porous polymers in this method. Optimization of the reaction system (composition of the mixed solvent, combination of the pore-generator) and reaction conditions (reaction temperature, monomer concentration, amount of the catalyst used) should be necessary for the effective synthesis of the porous polymers.

**Scheme 1:**
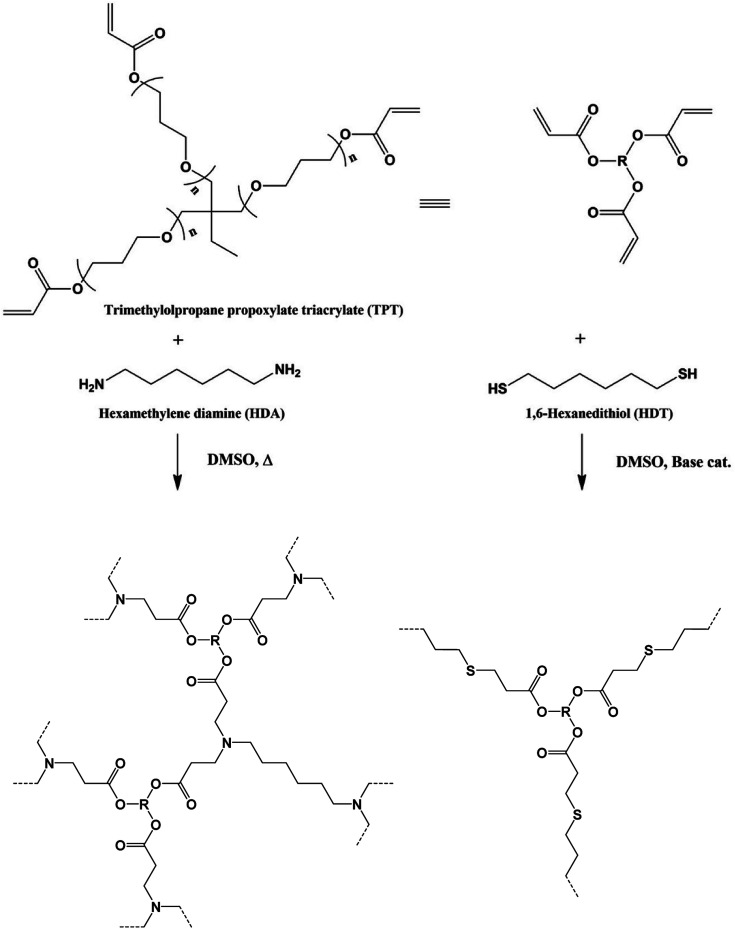
Synthesis mechanism of gels and porous polymers by means of Michael addition reaction of multi-functional acrylate compound, TPT, and HDA, HDT in DMSO.

Previously, we have developed various gels, which are synthesized by addition reactions between multi-functional compounds and α,ω-bifunctional compounds in various solvents, for example, hydrosilylation reaction of multifunctional siloxane or silsesquioxane and α,ω-diolefin, thiol-ene reaction of multifunctional thiol and diacrylate, multifunctional vinyl siloxane or silsesquioxane and dithiol, azide-alkene reaction of multifunctional vinyl siloxane or silsesquioxane and diazide, and so on.^6^ This molecular design makes it possible to synthesize the gels with homogeneous, controlled mesh size, and designed molecular geometry network structures. In a series of the studies, we found that the reactions of multi-functional thiol and diisocyanate compounds in toluene induced phase separation, which yielded porous polymers without phase separator.^6*e*^ We have been studying the affinity between the network structure and solvent in the gels synthesized by addition reactions between multi-functional compounds and α,ω-bifunctional compounds, and found Michael addition reactions of conventional multifunctional acrylate compound trimethylolpropane propoxylate triacrylate (TPT) with hexamethylene diamine (HDA) or 1,6-hexanedithiol (HDT) in dimethyl sulfoxide (DMSO) successfully yielded the porous polymers. These reaction systems make simplify the post-processing of the porous polymers. Here we report the effect of the polymerization conditions, polymerization temperature, monomer feed ratio, and monomer concentration, on the production state and structure of the TPT–HDA, -HDT polymers. The porous structure, space structure and size, can be precisely controlled by the reaction conditions. The mechanical property and absorption capacity of the porous polymer were quantitatively evaluated to study the effect of the porous structure on the features. We also report the coloration of the TPT–HDA porous polymer in DMSO, in the as prepared state, due to structural coloration, so called Christiansen filter effect. Transition of the color was observed depended on the observation temperatures (Scheme 1).

## Experimental

### Materials

Trimethylolpropane propoxylate triacrylate (TPT, average *M*_n_ 644, Sigma-Aldrich Co., LLC.), was commercially obtained, and were purified by passing through Al_2_O_3_ column before use to remove a polymerization inhibitor. Hexamethylene diamine (HDA), 1,6-hexanedithiol (HDT), and dimethyl sulfoxide (DMSO) were purchased from Kanto Chemical Co., Inc., and used without further purification. Triethylamine (TEA, Kanto Chemical Co., Inc.), piperidine (Pip, Kanto Chemical Co., Inc.), and (*E*)-1-piperidino-3-(2-hydroxyphenyl)-2-propen-1-one (PipHPP, Wako Pure Chemical Industries), were commercially obtained, and used as received.

### Synthesis of porous polymers

#### Synthesis of TPT–HDA porous polymers

The reaction of TPT with HDA, [TPT]/[HDA]: 4/3 (mol mol^−1^), monomer concentration in the reaction solution: 20 wt%, is described as an example. TPT (0.77 g, 1.2 mmol), HDA (0.105 g, 0.9 mmol), and DMSO (3.2 mL), and were added to a 10 mL of glass ample tube (diameter 13 mm). The reaction system was stirred by vortex mixer for several minutes. After the sample tube was sealed by burning off, the reaction system was heated at the desired temperature for 24 h. The obtained porous polymer was washed by excess of methanol with ultrasonification for several time, and dried *in vacuo* at room temperature for 6 h. The reactions with different [TPT]/[HDA] feed ratio and/or monomer concentration were conducted with the same procedures.

#### Synthesis of TPT–HDT porous polymers

The reaction of TPT with HDT, [TPT]/[HDT]: 2/3 (mol mol^−1^), monomer concentration in the reaction solution: 25 wt%, is described as an example. TPT (0.64 g, 1.0 mmol), DMSO (2.3 mL), and TEA (30 μL) were added to a 10 mL of glass ample tube (diameter 13 mm). The reaction system was stirred by vortex mixer to make the homogeneous solution. After HDT (0.225 mL, 1.5 mmol) was added, the reaction system was stirred immediately. The polymer was obtained within ∼1 min, and the reaction system was kept at room temperature for 24 h. The obtained porous polymer was washed by excess of methanol with ultrasonification for several times, and dried *in vacuo*. At room temperature for 6 h. The reactions with different [TPT]/[HDT] feed ratio and/or monomer concentration were conducted with the same procedures.

### Analytical procedures

FT-IR spectra of the reaction solutions and polymers were recorded on a Jasco FT/IR-410 (JASCO Corporation). The samples were prepared between KBr-Real Crystal IR-Card and Slip (International Crystal Laboratories), and 30 scans were accumulated from 4000 to 500 cm^−1^.

Scanning electron microscopy (SEM) images of the porous polymers were obtained by a JEOL JSM-7610F microscope with an LEI detector at an acceleration voltage of 3.0 kV.

Surface area of the porous polymers were measured by nitrogen sorption using an Autosorb 6AG (Quantachrome), and determined by Brunauer–Emmett–Teller (BET) equation.

Mechanical properties of the porous polymers were investigated by compression test with an universal testing instrument, Tensilon RTE-1210 (ORIENTEC Co. LTD.). The test samples were cut to 1 cm cube, and pressed at a rate of 0.5 mm min^−1^ at room temperature. Young's modulus was determined from slope of the stress–strain curve acquired by the compression test in the elastic deformation region (at 10% of strain).

Differential scanning calorimetry (DSC) measurements of the porous polymers were conducted with a Rigaku DSC 8230. The transition temperature was determined on the 2^nd^ heating process from −50 °C to 200 °C at a rate of 10 °C min.

UV-vis spectroscopy of the porous polymers prepared in DMSO was conducted with a SHIMADZU UV-1600PC, and the transmittance was recorded by 0.1 nm at a scan rate of 550 nm min^−1^.

## Results and discussion

### TPT–HDA porous polymer

**Fig. 1:**
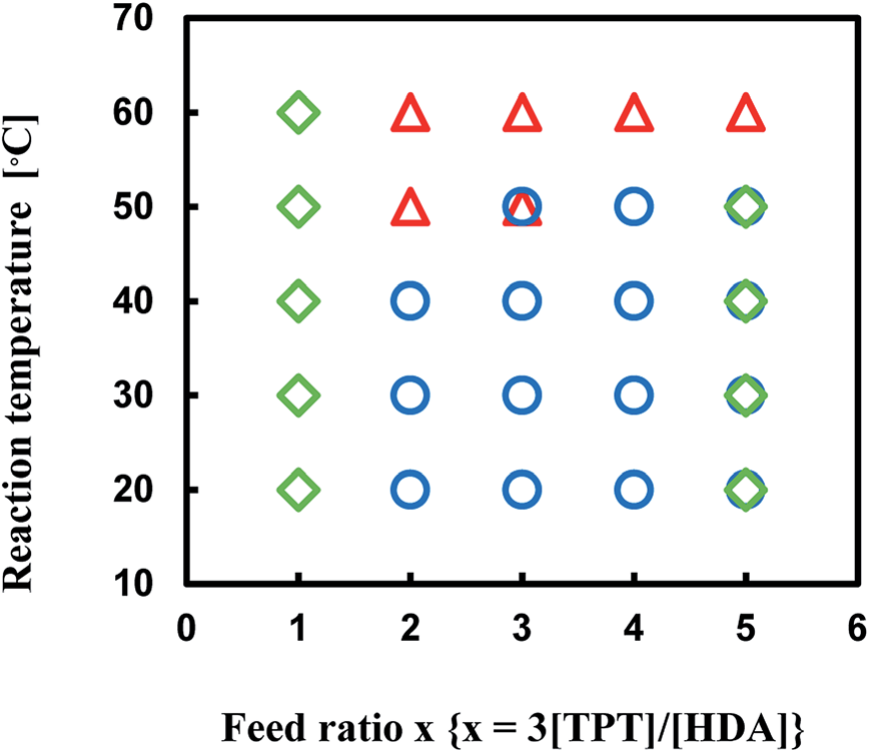
Production diagram of the TPT–HDA system, ○ porous polymer, △ gel, ◇ solution, monomer concentration: 20 wt%, overlap means co-existence of two types of polymers.

The reaction of TPT and HDA was conducted under various conditions, temperature, [TPT]/[HDA] feed ratio, and monomer concentrations in DMSO. The conditions strongly affected the state (porous polymer, gel, solution) of the reaction system. Fig. 1 shows production diagram of the TPT–HDA system with 20 wt% of monomer concentration. The reactions at the temperatures ranged from 20 to 40 °C with the [TPT]/[HDA] feed ratio of 2/3–4/3 (mol mol^−1^) preferentially formed porous polymers. The reaction systems with the [TPT]/[HDA] feed ratio of 1/3 or 8/3 (mol mol^−1^) formed viscous solution. Non-equivalent feed of acrylate and amine should not be suitable to form the network structure. The gelation (network formation) rate should be higher than that of the phase separation rate at 60 °C, and the reactions preferentially formed gels. In the case of the reactions at 50 °C, the reactions with the [TPT]/[HDA] feed ratio of 4/3 (mol mol^−1^) formed porous polymer. The [TPT]/[HDA] feed ratio of 4/3 (mol mol^−1^) means equivalent amount of acrylate and NH groups in the reaction system, and should form network structure effectively accompanied by the phase separation.

**Fig. 2:**
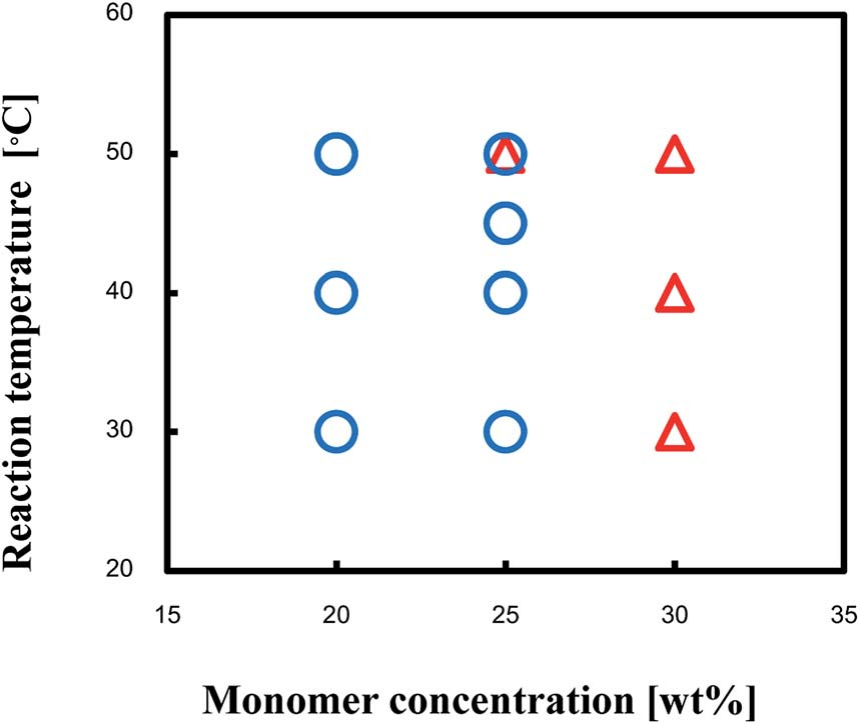
Production diagram of the TPT–HDA system, ○ porous polymer, △ gel, [TPT]/[HDA] = 4/3 (mol mol^−1^), solvent: DMSO, overlap means co-existence of two types of polymers.

Production diagram of the TPT–HDA system with the [TPT]/[HDA] feed ratio of 4/3 (mol mol^−1^) at 30–50 °C is illustrated in Fig. 2 to show the effect of monomer concentration. The reaction systems with 20 and 25 wt% of monomer concentrations yielded the porous polymers. By contrast, the reactions with 30 wt% of monomer concentration yielded the gels independent of the reaction temperatures. The increase of the monomer concentration would change the state from separated two-phase to single-phase.

Reaction of TPT and HDA in DMSO was traced by FT-IR spectroscopy (ESI Fig. 1). Intensity of the absorption peaks at around 1650 cm^−1^, 810 cm^−1^, or 1410 cm^−1^ derived from vibration of primary or secondary amine, C=C bending of acryloyl group, or CH_2_ bending of acryloyl group decreased after the reaction, indicating promotion of aza-Micahel reaction between acrylate and amine groups. Reaction conversions in TPT–HDA gel and porous polymer, [TPT]/[HDA] feed ratio of 4/3 (mol mol^−1^), evaluated from the peak intensity of the acrolyl groups in FT-IR spectra were 59% (monomer concentration: 20 wt% at 60 °C) and 64% (monomer concentration: 25 wt% at 45 °C), respectively. These results indicate that the reaction conversions are high enough to form the network structure independent of the production state.

**Fig. 3:**
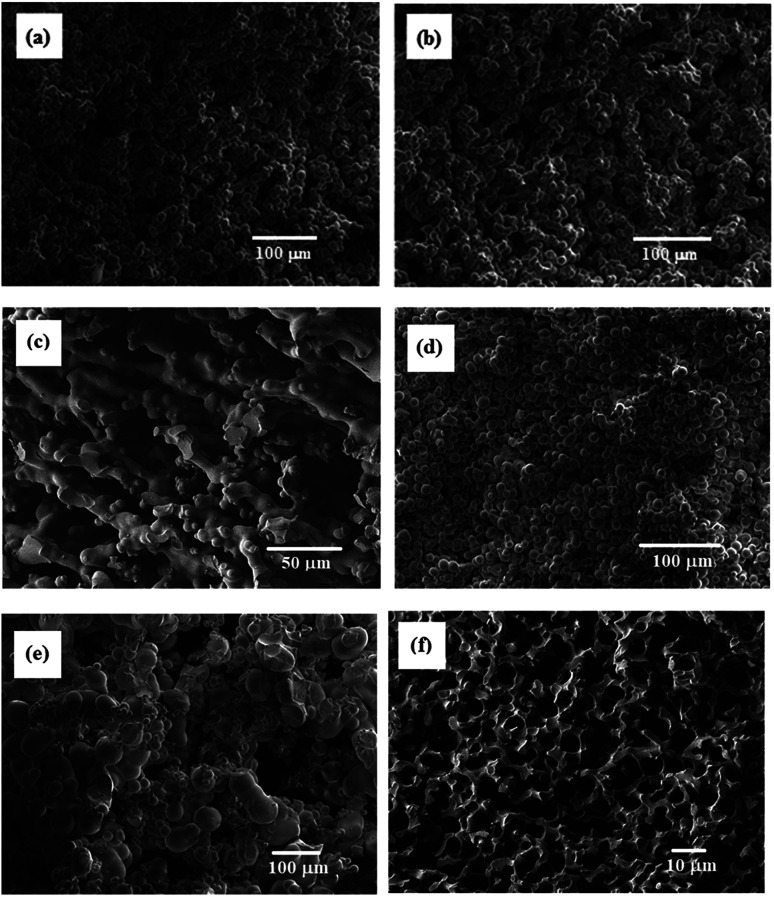
SEM images of TPT–HDA porous polymers, [TPT]/[HDA] = 4/3 (mol mol^−1^), monomer concentration in the reaction solution: 20 wt% at (a) 40 °C, (b) 45 °C, and (c) 50 °C, monomer concentration in the reaction solution: 25 wt% at (d) 30 °C, (e) 40 °C, and (f) 45 °C.

SEM images of some resulting porous polymers, which were obtained in the reactions with [TPT]/[HDA] feed ratio of 4/3 (mol mol^−1^) are shown in Fig. 3. The reaction with 20 wt% of monomer concentration at 30 and 40 °C yielded the porous structure formed by aggregated globules of ∼5 μm diameter, as shown in Fig. 3(a) and (b). Continuous structure formed by broadly connected globules was observed in the porous polymer obtained in the corresponding reaction at 50 °C (Fig. 3 (c)). The reaction with 25 wt% of monomer concentration at 30 °C produced the porous structure formed by aggregated globules of ∼5 μm diameter (Fig. 3(d)). The porous structure formed by aggregated globules with relatively large, 50 μm, and small, 5–10 μm, sizes was observed in the porous polymer obtained in the corresponding reaction at 40 °C (Fig. 3(e)). Furthermore, the reaction at 45 °C yielded the porous polymer with co-continuous monolithic structure as shown in Fig. 3(f). Variation of the porous structures can be explained by spinodal decomposition during the network formation. When the reaction rate is high at high reaction temperature, the co-contentious monolithic structure is fixed at the early stage of the spinodal decomposition. In the case of the low temperature reactions, the secondary phase separation, which forms globules at later stage of the spinodal decomposition, should be induced during the reaction due to the low reaction rate. The similar results were reported in other reaction systems.^7^

Surface area of the porous polymers were relatively small, less than 2 m^2^ g^−1^, and could not be determined quantitatively by BET method. The small surface area of the porous polymers should be derived from the macroporous structure.

**Fig. 4:**
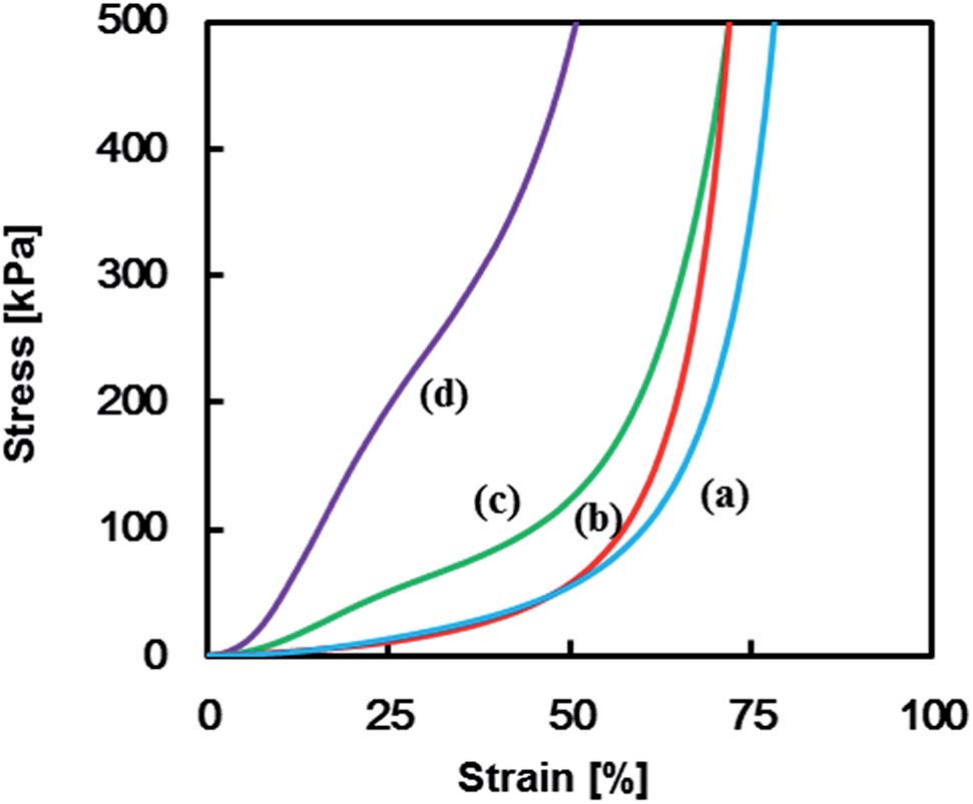
Stress–strain curves of TPT–HDA porous polymers, [TPT]/[HDA] = 4/3 (mol mol^−1^), monomer concentration in reaction solution: 25 wt%, reaction temperature: (a) 40 °C, (b) 44 °C, (c) 44.5 °C, and (d) 45 °C.

Mechanical properties of the TPT–HDA porous polymers were investigated by the compression test. The porous structure of the test samples was precisely controlled by the reaction temperatures ranged from 40–45 °C in the reactions of [TPT]/[HDA] = 4/3 (mol mol^−1^), monomer concentration: 25 wt% based on the results in Fig. 3 (ESI Fig. S2†). Fig. 4 shows stress–strain curves of the TPT–HDA porous polymers, and the results are summarized in Table 1. All the porous polymers were unbreakable by the compression test under 50 N. The glass transition temperature of a TPT–HDA porous polymer prepared from the reaction solution with 25 wt% monomer at 45 °C is −24.4 °C, and flexible feature in the compression test at room temperature is derived from amorphous phase of the porous polymer. The porous polymers with monolithic structure showed hard property, and the Young's modulus decreased with increasing of the size of co-continuous structure. Profiles of the stress–strain curves showed S-curve, indicating two steps deformation. The former or latter deformation should be derived from compression of contentious holes or pressed bulk. The soft feature of the porous polymers with large co-continuous structure should be caused by former deformation with low Young's modulus. The porous polymers formed by aggregated globules showed lower Young's modulus than that of the porous polymers formed by monolithic structure. Dissociation of the aggregation would be the driving force of the initial deformation of these porous polymers.

**Table 1:** Mechanical properties of dried TPT–HDA porous polymers^a^

Reaction temperature [°C]	Young's modulus^b^ [kPa]	Porous structure (SEM)	Size^c^ [mm]
40	17.8	Globule (Fig. 3(e))	18, 55
44	18.8	Globule (Fig. S2(a))	13
44.5	52.4	Monolith (Fig. S2(b))	19
45	80.9	Monolith (Fig. 3(f))	5.7

a [TPT]/[HDA] = 4/3 (mol mol^−1^), monomer concentration in reaction solution: 25 wt%.

b Not breakable under 50 N.

c Average diameters of the globules or continuous holes of monolith determined by image processing.

The TPT–HDA porous polymer absorbs various organic solvents, chloroform, DMSO, dimethyl formamide, ethanol, and so on. The absorption of chloroform and DMSO was especially quick, and was completed within 10 min. The absorption of chloroform and DMSO was quantitatively investigated by TPT–HDA porous polymers formed by the aggregated globules or monolithic structure, prepared from the reaction solutions with 20 wt% or 25 wt%, respectively, at 45 °C (Table 2). Both the weight gain and volume gain in chloroform absorption were higher than those in DMSO. The calculated solubility parameters of the repeating unit of TPT–HDA porous polymers is 17.3 (MJ m^−3^)^1/2^.^8^ The solubility parameters of chloroform and DMSO are 19.0 and 29.7 (MJ m^−3^)^1/2^. Close solubility parameters between the polymer and solvent is an index of high miscibility. The values of the solubility parameters indicate that miscibility of the porous polymer with chloroform is much higher than that with DMSO, which should induce higher absorption of chloroform. Porous structure also affected the absorption capacity, and the polymer with aggregated globules showed larger weight gain than that with monolithic structure. By contrast, there was no difference in volume gain. One explanation of these results are that the porous polymer with aggregated globules would absorb the solvents not only in the vacant space and but inside of the globules.

**Table 2:** Absorption properties of dried TPT–HDA porous polymers a

Monomer concentration [wt%]	Solvent	Weight gain [wt%]	Volume gain [vol%]	Porous structure (SEM)
20	CHCl_3_	1610	324	Globule (Fig. 3(b))
25	CHCl_3_	934	331	Monolith (Fig. 3(f))
20	DMSO	519	135	Globule (Fig. 3(b))
25	DMSO	298	140	Monolith (Fig. 3(f))

a [TPT]/[HDA] = 4/3 (mol mol^−1^), monomer concentration in reaction solution: 20 wt%, 25 wt%, reaction temperature: 45 °C, reaction time 48 h.

**Fig. 5:**
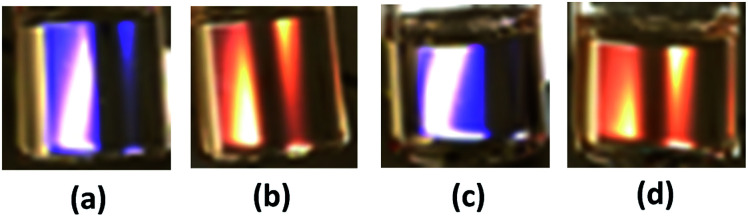
Photos of TPT–HDA porus polymers in DMSO (as prepared state in ample tube), monomer concentration of preparation: (a and b) 20 wt%, (c and d) 25 wt%, reaction time: (a and c) 24 h, (b and d) 48 h, reaction temperature: 45 °C, observation temperature: room temp.

**Fig. 6:**
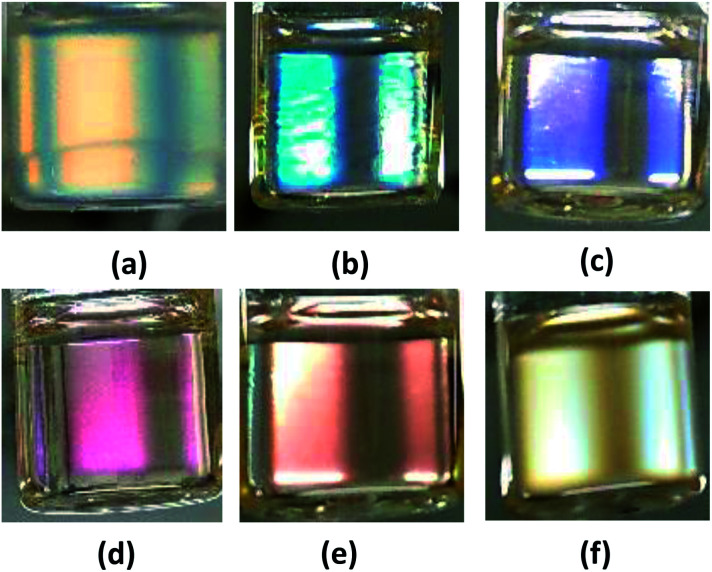
Photos of a TPT–HDA porous polymer in DMSO (as prepared state in ample tube), monomer concentration of preparation: 25 wt%, reaction time: 48 h, reaction temperature: 45 °C, observation temperature: (a) 5 °C, (b) 20 °C, (c) 30 °C, (d) 40 °C, (e) 45 °C, and (f) 60 °C.

The TPT–HDA porous polymers with chloroform were whitely clouded. By contrast, those with DMSO turned clear, and slightly colored under the white light (ESI Fig. S3 and S4†). The coloring feature of the TPT–HDA porous polymer in DMSO is derived from not absorption of light due to electron excitation (like dyes and pigments) but periodic microstructures, so called structural coloration. In the case of the present TPT–HDA system, the porous polymer size is much larger than the correlation of wavelength of the visible light. The diffusion reflection of irradiated visible light should would occur in the systems, and the systems appears white when the reflective indexes of polymer and solvent are different each other, as observed in TPT–HDA with chloroform. By contrast, when the reflective indexes of polymer and solvent are closed, the visible light transmits through the system, as observed in TPT–HDA with DMSO. We focused on the coloring behaviour of the porous polymers as prepared in DMSO. Effect of the reaction time on the coloring of the porous polymers was studied. Fig. 5 shows the TPT–HDA porous polymer as prepared in DMSO, [TPT]/[HDA] = 4/3 (mol mol^−1^), monomer concentration: 20 wt% (connected globules structure) or 25 wt% (monolithic structure). The porous polymers obtained with 24 h or 48 h reactions at 45 °C colored slightly purple or orange, respectively, independent of the monomer concentration. Reaction conversion would affect the color of the porous polymers. Effect of the observation temperatures on the coloring of TPT–HDA porous polymer, [TPT]/[HDA] = 4/3 (mol mol^−1^), monomer concentration: 25 wt%, are demonstrated in Fig. 6. The color changed from blue, purple, pink, orange, to yellow with increasing of the observation temperature from 5 °C to 60 °C on the heating process. The color did not change (stayed yellow) at the temperatures higher than 60 °C. The sample showed reverse color change on the cooling process. These transitions in the transmittance spectra are derived from Christiansen filter effect, as reported in some porous gels, dispersions, and colloids in solvents.^9^

**Fig. 7:**
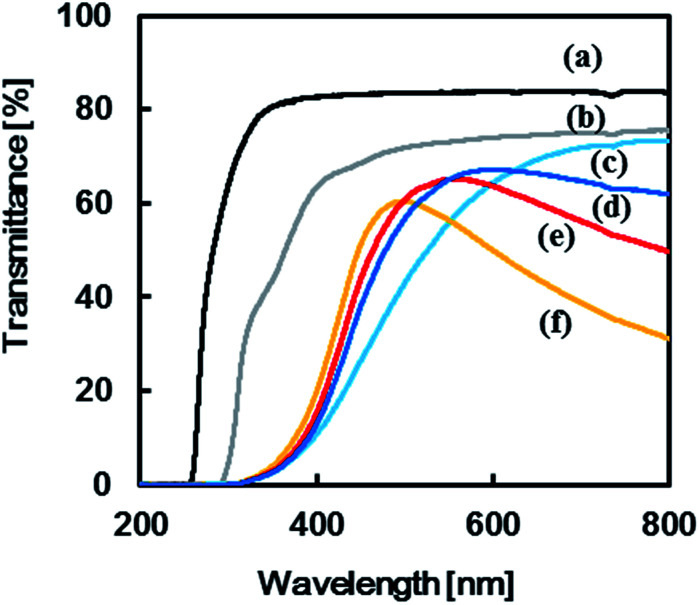
Transmission spectra of (a) DMSO, (b) reaction solution and TPT–HDA porous polymer, monomer concentration: 25 wt%, prepared at 40 °C, reaction time: (c) 24 h, (d–f) 48 h, observation temperature: (a–d) room temperature, (e) 40 °C, (f) 60 °C.

Transmittance spectra of the TPT–HDA porous polymers, monomer concentration: 25 wt% prepared at 40 °C, are shown in Fig. 7. The reaction time to prepare the porous polymer affected profile of the transmittance spectra. The porous polymer obtained with 48 h reaction showed lower transmittance at long wave length, more than 600 nm, in comparison with the polymer obtained with 24 h reaction. The increase of the measured temperature decreased the transmittance at the long wave length accompanied by blue-shift of peak maxima of the transmittance peak, from 590 nm to 490 nm. The color of the TPT–HDA porous polymers in DMSO should be derived from both the reflected light and transmitted light. The broader perspective red-shift of the wave length of the visual aspect with increasing of the observation temperature should be derived from the decrease of the transmittance at long wave length of the reflected light. By contrast, the localized blue-shift of the visual aspect with increasing of the measured temperature should be derived from the blue-shift of the transmitted light. The combination of these transitions in both the reflected light and transmitted light *via* the TPT–HDA porous polymers in DMSO would cause the discontinuous wave length of the visual aspect.

### TPT–HDT porous polymers

**Fig. 8:**
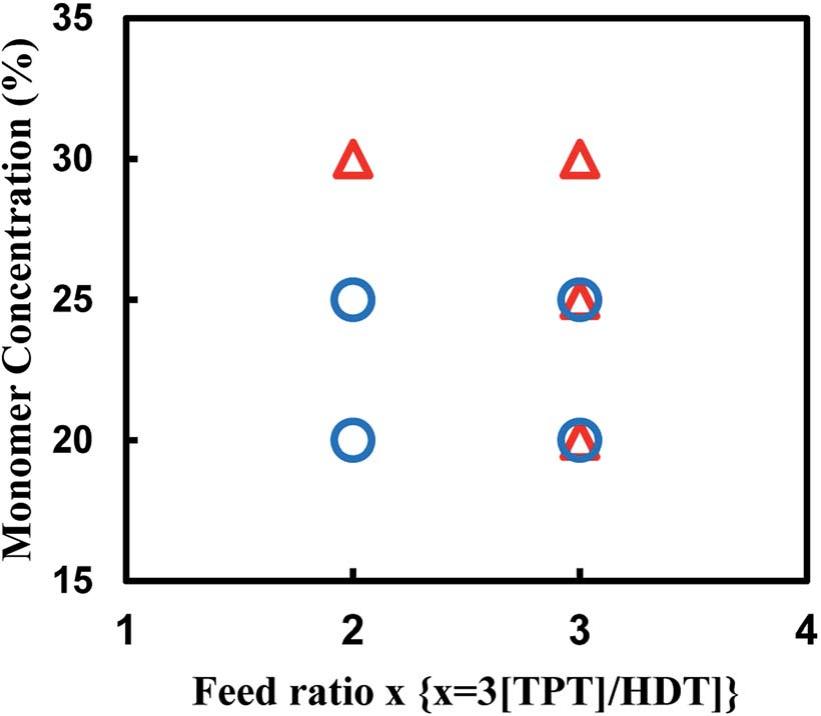
Production diagram of the TPT–HDT system, ○ porous polymer, △ gel, reaction temperature: room temp., reaction time: 48 h, overlap means co-existence of two types of polymers.

We also attempted to synthesize the porous polymer from multifunctional acrylate and dithiol using thio-Michael addition reaction in some organic solvents, and found the reaction of TPT and HDT in DMSO formed porous polymer. The reaction of TPT and HDT was conducted under various conditions; [TPT]/[HDT] feed ratio, and monomer concentrations in DMSO at room temperature using TEA as the base catalyst. Fig. 8 shows production diagram of the TPT–HDT system. The reactions with the [TPT]/[HDT] feed ratio of 2/3 (mol mol^−1^) and 20 or 25 wt% of monomer concentration preferentially formed the porous polymers. The reactions with the [TPT]/[HDT] feed ratio of 1/1 (mol mol^−1^) yielded mixture of the porous polymer and gel. Inequivalent feed of acrylate and SH would form imperfect network, which induced heterogeneous state of the resulting systems. Homogeneous gels were obtained in the reaction with 30 wt% of monomer concentration. Higher monomer concentration would change the state from two-phase separation to single-phase.

The molecular structure of the porous polymers prepared by TEA, Pip, and PipHPP as the base catalysts was studied by FT-IR spectroscopy (ESI Fig. S5†). The absorption peak at around 2550 cm^−1^ derived from stretching of thiol group was almost disappeared, and intensity of the absorption peaks at 810 cm^−1^, or 1410 cm^−1^ derived from C=C bending of acryloyl group, or CH_2_ bending of acryloyl group decreased in comparison with the spectrum of TPT, indicating almost complete promotion of Michael reaction between acrylate and thiol groups with all the catalysts.

**Fig. 9:**
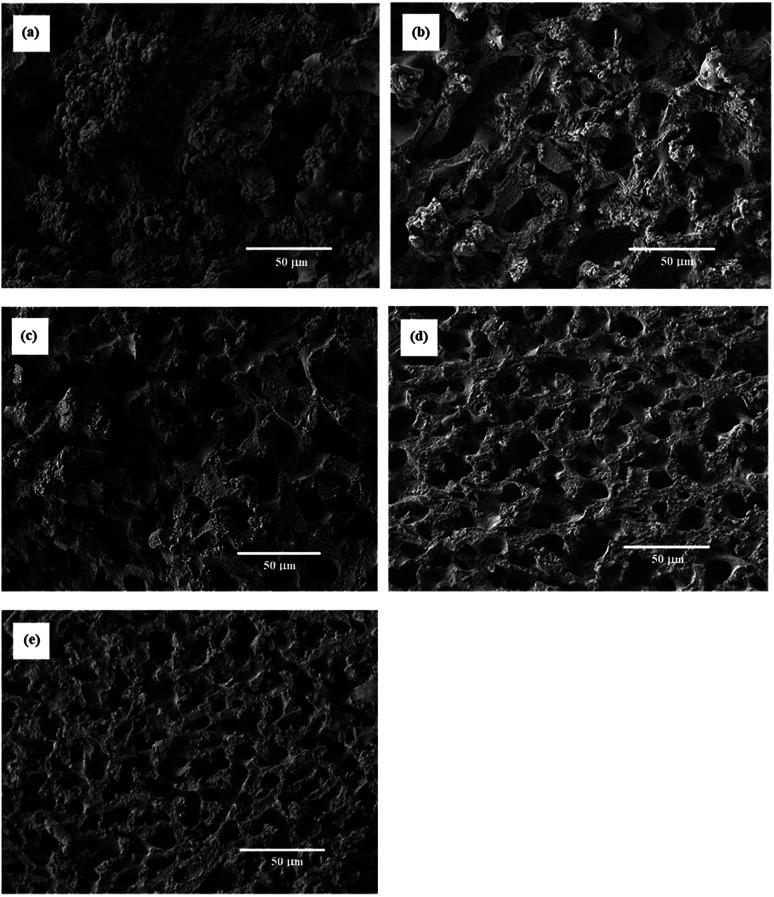
SEM images of TPT–HDT porous polymers, [TPT]/[HDT] = 2/3 (mol mol^−1^), monomer concentration in reaction solution: (a) 23 wt%, (b) 23.5 wt%, (c) 24.5 wt%, (d) 25 wt%, (e) 25.5 wt%, catalyst (TEA) concentration in reaction solution: 70.7 μmol mL^−1^, reaction temperature: r.t., reaction time: 48 h.

Effect of the monomer concentration on the porous structure of the resulting porous polymers was precisely studied in the reactions with [TPT]/[HDT] feed ratio of 4/3 (mol mol^−1^). SEM images of some resulting porous polymers, which were obtained in the various monomer concentrations, are shown in Fig. 9. The monomer concentrations were sensitive to the porous structure. The reaction with 23 wt% of monomer concentration yielded the porous structure formed by aggregated globules about 1–5 μm diameter and deformed large continuous structure, as shown in Fig. 9(a). By contrast, co-continuous structure was observed in the porous polymers obtained in the corresponding reactions with 23.5, 24.5, and 25.5 wt% of monomer concentrations, as shown in Fig. 9(c)–(e), respectively. The increase of the monomer concentration decreased size of the co-continuous structure. The reaction with higher monomer concentration increased the reaction rate, and the co-contentious monolithic structure should be fixed at the early stage of the spinodal decomposition.

**Fig. 10:**
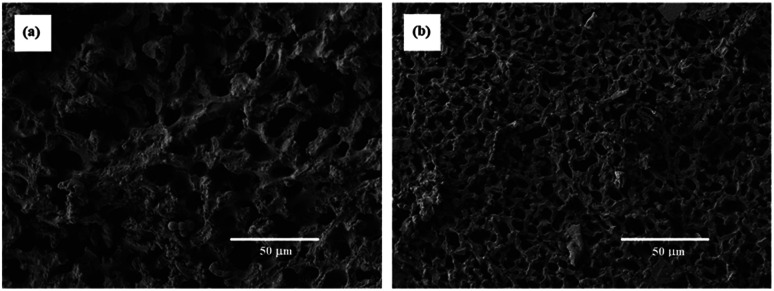
SEM images of TPT–HDT porous polymers, [TPT]/[HDT] = 2/3 (mol mol^−1^), monomer concentration in reaction solution: 25 wt%, catalyst (Pip) concentration in reaction solution: (a) 1.66 μmol mL^−1^, (b) 3.33 μmol mL^−1^, reaction temperature: r.t., reaction time: 24 h.

The SEM images of the porous polymers obtained from the reaction systems with [TPT]/[HDT] feed ratio of 2/3 (mol mol^−1^) and 25 wt% of monomer concentration using Pip as the base catalyst are shown in Fig. 10 (a) and (b). The porous polymers showed co-contentious structure with the isolated micro holes, as observed in the corresponding polymers obtained using TEA as the catalyst, as shown in Fig. 9(d). The increase of the Pip concentration decreased the size of the co-contentious structure. The increase of the reaction rate should fix the co-contentious monolithic structure at the early stage of the spinodal decomposition.

**Fig. 11:**
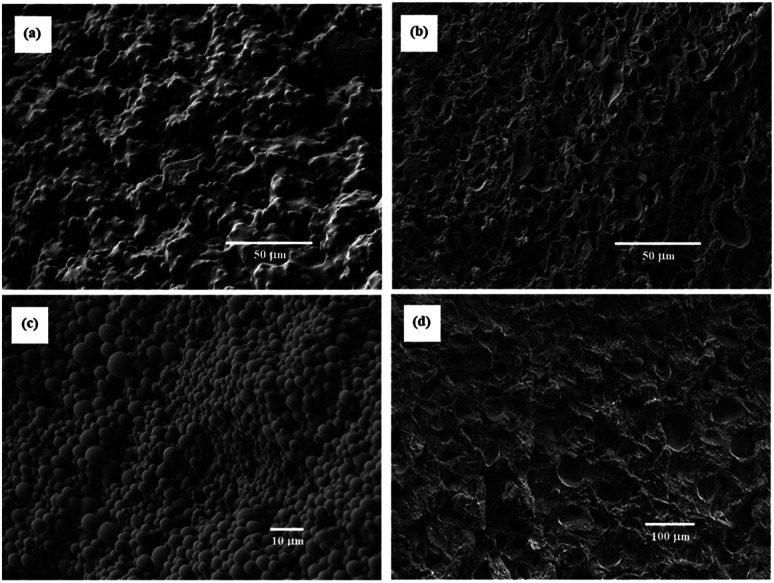
SEM images of TPT–HDT porous polymers, [TPT]/[HDT] = 2/3 (mol mol^−1^), monomer concentration in reaction solution: 25 wt%, catalyst (PipHPP) concentration in reaction solution: (a) 0.427 μmol mL^−1^, (b) 1.42 μmol mL^−1^, (c) 1.71 μmol mL^−1^, reaction temperature: r.t., reaction time: 48 h.

A photobase generator, PipHPP, produces Pip by irradiation of UV. The application of the photobase generator makes it possible to yield the porous polymers by UV irradiation. The SEM images of the porous polymers obtained from the reaction systems with [TPT]/[HDT] feed ratio of 2/3 (mol mol^−1^) and 25 wt% of monomer concentration are shown in Fig. 11(a)–(d). The porous polymers obtained from low PipHPP system showed the structure formed by broadly connected globules, as shown in Fig. 11(a). The reaction systems with higher PipHPP concentration yielded the porous polymers formed by mixed structure of the co-contentious and isolated distorted holes, as shown in Fig. 11(b) and (c). The increase of the reaction rate with increasing of the PipHPP concentration may induce precipitation of the resulting polymer, which would disturb the porous structure derived from phase separation.

**Fig. 12:**
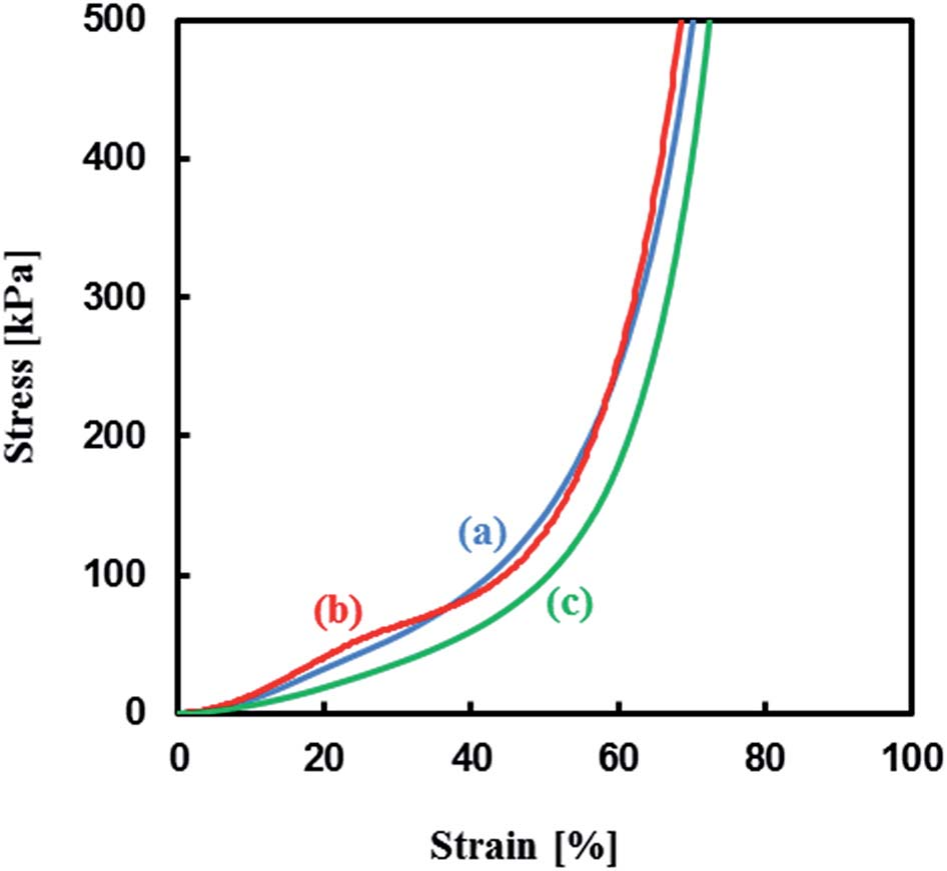
Stress–strain curves of TPT–HDT porous polymers, [TPT]/[HDT] = 2/3 (mol mol^−1^), monomer concentration in reaction solution: 25 wt%, catalyst concentration in reaction solution: (a) [TEA] = 70.7 μmol mL^−1^, (b) [Pip] = 1.66 μmol mL^−1^, (c) [PipHPP] = 1.42 μmol mL^−1^, reaction temperature: r.t., reaction time: 48 h.

Mechanical properties of the TPT–HDT monolithic porous polymers, where [TPT]/[HDT] = 2/3 (mol mol^−1^) and monomer concentration in reaction solution: 25 wt%, obtained with different catalysts, TEA, Pip, and PipHPP, were investigated. Fig. 12 shows the representative stress–strain curves of these polymers having co-continuous monolithic structure. All the porous polymers were unbreakable under the compressive load of 50 N and showed similar profiles in the stress–strain curves due to the same monomer concentration in the reaction solutions and similar porous structures. The values of the Young's modulus of the polymers are given in Table 3. The glass transition temperature of a TPT–HDT porous polymer prepared from the reaction solution with 25 wt% using Pip as the catalyst is −30.0 °C. The flexible feature, unbreakable and low Young's modulus, should be derived from amorphous phase of the porous polymer.

**Table 3:** Mechanical and absorption properties of dried TPT–HDT monolithic porous polymers^a^

Catalyst (SEM Fig.)	Young's modulus^b^ [kPa]	Solvent	Weight gain [wt%]	Volume gain [vol%]
TEA (9(d))	49.6	CHCl_3_	2220	771
		DMSO	208	137
Pip (10(a))	53.4	CHCl_3_	1930	832
		DMSO	238	124
PipHPP (11(b))	36.4	CHCl_3_	2160	930
		DMSO	214	126

a [TPT]/[HDT] = 2/3 (mol mol^−1^), solvent of reaction system: DMSO, monomer concentration in reaction solution: 25 wt%, catalyst concentration in reaction solution: [TEA] = 70.7 μmol mL^−1^, [Pip] = 1.66 μmol mL^−1^, [PipHPP] = 1.42 μmol mL^−1^, reaction temperature: r.t., reaction time: 48 h, porous structure: monolith.

b Not breakable under 50 N.

The absorption of chloroform and DMSO was quantitatively investigated by TPT–HDT porous polymers formed by the monolithic structure, prepared from the reaction solutions with 25 wt% of monomer concentration using different base catalysts. Theses porous polymers absorbed about 2000 wt% of chloroform and 200 wt% of DMSO. The TPT–HDT porous polymers absorbed much chloroform in comparison with the TPT–HDA porous polymers, as summarized in Table 3. One possibility to explain these results is the miscibility of the network structure of the porous polymers and solvents. The calculated solubility parameters of the repeating unit of TPT–HDA and TPT–HDT porous polymers are 17.3 and 16.4 (MJ m^−3^)^1/2^,^8^ and the solubility parameter of chloroform is 19.0 (MJ m^−3^)^1/2^. Close solubility parameters between the polymer and solvent would show high miscibility. If absorbance capacity of the porous polymer obeys this theory, the TPT–HDA porous polymer should absorb more chloroform than the TPT–HDT porous polymer. The contrary results to the theory would be derived from difference in the porous structure. Thickness of the framework of the TPT–HDA porous polymers, 1–3 μm as shown in Fig. 3(f), was smaller than that of the TPT–HDT porous polymers used in the experiment, 5–15 μm as shown in Fig. 9(d), and showed low absorbance capacity of chloroform with the TPT–HDA porous polymers.

The TPT–HDT porous polymers did not show Christiansen filter effect (ESI Fig. S6†). The small difference in chemical structure, NH or S, should make difference in refractive index between the TPT–HDA and TPT–HDT porous polymers.

## Conclusions

Michael reaction of multifunctional acrylate and bifunctional compounds have been investigated to synthesize porous polymers induced by the phase separation. Aza-Michael addition reaction of TPT and HDA in DMSO successfully yielded the porous polymer under the conditions of [TPT]/[HDA]: 2/3–3/5 (mol mol^−1^), monomer concentration: 20–25 wt%, and reaction temperature: 20–50 °C. The porous structure was controlled by reaction conditions, and formed connected globules and co-continuous monolithic structure. The porous polymers with monolithic structure showed higher Young's modulus than those with connected globules. The TPT–HDA porous polymer absorbed various solvents, especially CHCl_3_. The porous polymers in DMSO as prepared state were colored depending on the reaction time and the observation temperature induced by Christiansen filter effect. Thio-Michael reaction of TPT–HDT in DMSO in the presence of base catalysts also yielded the porous polymer successfully under the conditions of [TPT]/[HDT]: 2/3 (mol mol^−1^), monomer concentration: 20–25 wt%, at room temperature. The porous structure could be controlled by the catalysts amount of a photo base catalyst of PipHPP. The Young's modulus values of the TPT–HDT porous polymers were same level those of the TPT–HDA porous polymers. The TPT–HDT porous polymer also absorbed various solvents, but did was colored in DMSO.

As mentioned above, aza- and thio-Michael addition reactions of the multi-functional acrylate, TPT, with conventional diamine, HDA, or dithiol, HDT, in DMSO yielded the porous polymers in facile process. One of the merits of the both the present systems is that the porous polymers can be obtained without phase separator. This makes simplify the post-processing of the porous polymers. The reaction of TPT with HDA proceeds without catalyst. The reaction of TPT with HDT can be promoted by a photo-base catalyst. The photo initiated polymerization should make it possible to patter and 3D print of the TPT–HDT porous polymers. The reaction of the multi-functional molecules and bi-functional molecules in selected solvents must be useful method to develop the porous polymers induced by the phase separation during the polymerization. As the next step, we are developing new porous polymers by addition reaction of various multi-functional molecules with bi-functional molecules in conventional solvents. We are also trying to apply the present porous polymers for separation columns or catalyst supports. The coloring feature depended on the temperature of the TPT–HDA porous polymer in DMSO would be applicable to sensors. These results will be reported elsewhere.

## Conflicts of interest

There are no conflicts to declare.

## Supplementary Material

RA-010-C9RA09684A-s001
