# Supplementary material for: Synthesis and properties of porous polymers synthesized by Michael addition reactions of multi-functional acrylate, diamine, and dithiol compounds
Source: RSC Adv. 2019 Dec 23;10(1):60–9. doi: 10.1039/c9ra09684a (PMC9047082; doi:10.1039/c9ra09684a)
Supplement: RA-010-C9RA09684A-s001 [file RA-010-C9RA09684A-s001.pdf]

Electronic Supplementary Material (ESI) for RSC Advances.
This journal is © The Royal Society of Chemistry 2019

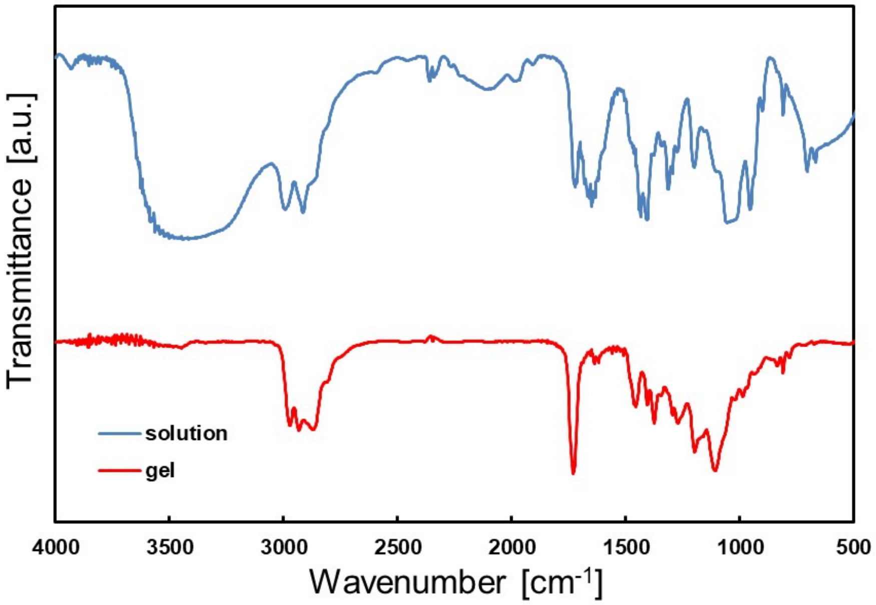


Figure S1 (a) FT-IR spectra of TPT-HDA system, before reaction (blue line), after reaction (red line), solvent: DMSO, monomer concentration: 20 wt%, reaction temperature: 60 $^{o}$C.

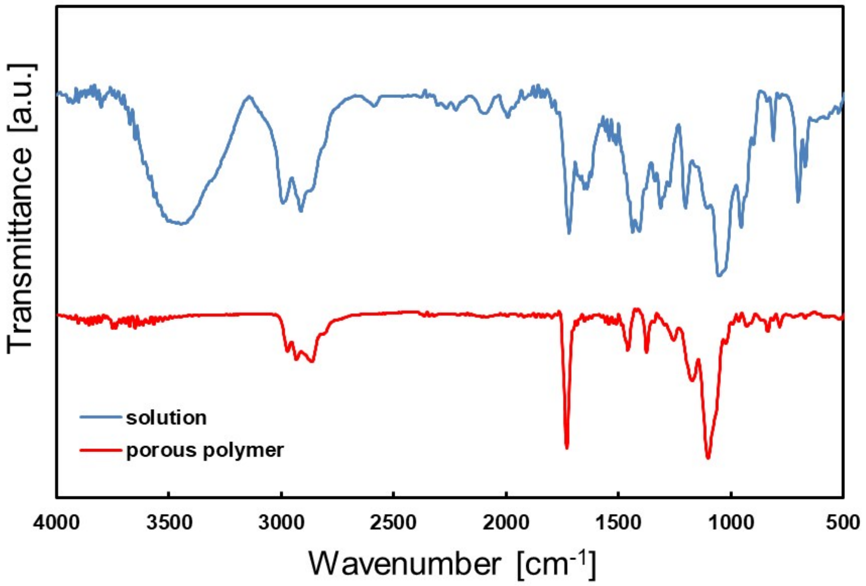


Figure S1 (b) FT-IR spectra of TPT-HDA system, before reaction (blue line), after reaction (red line), solvent: DMSO, monomer concentration: 25 wt%, reaction temperature: 45 $^{o}$C.

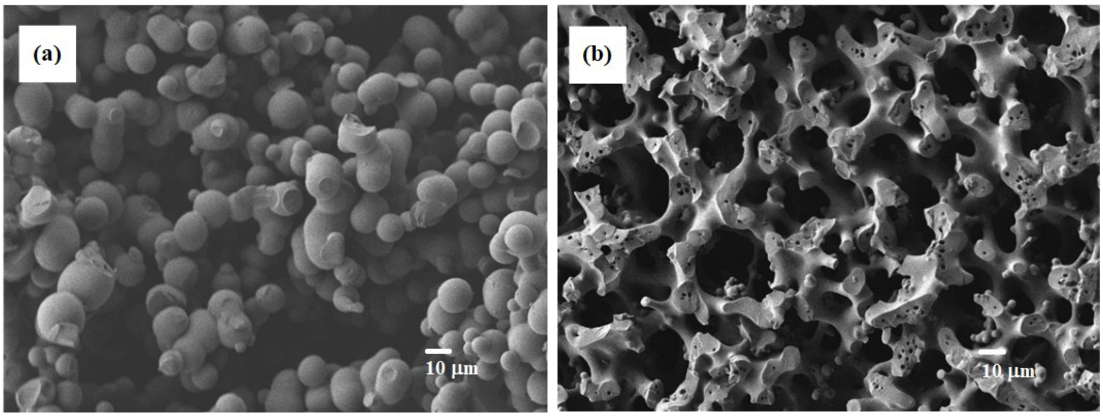


Figure S2 SEM images of TPT-HDA porous polymers used in compression test, monomer concentration in reaction solution: 25 wt%, reaction temperature: (a) 44 $^{o}$C, and (b) 44.5 $^{o}$C.

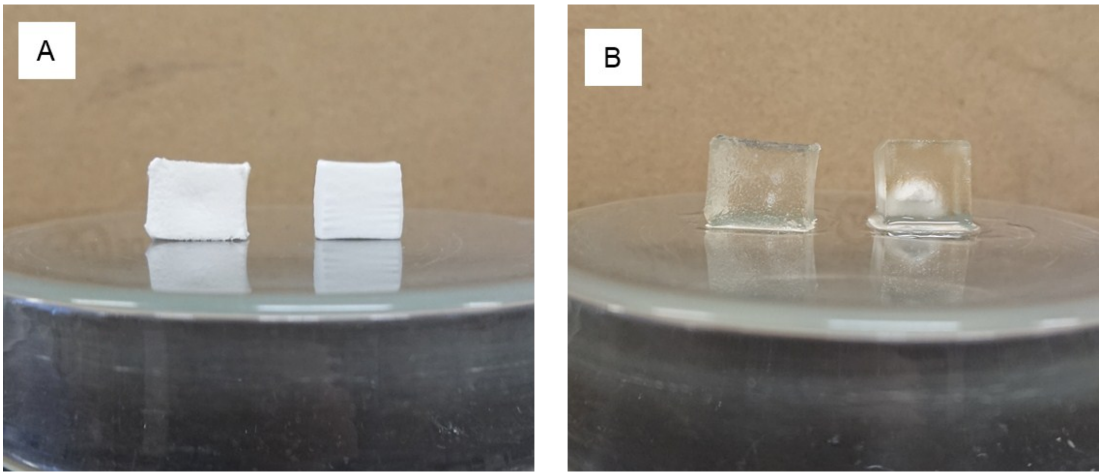


Figure S3 Photos of TPT-HDA porous polymers, (A) dried state, (B) dipped in DMSO, [TPT]/[HDA] = 4/3 (mol/mol), solvent of reaction system: DMSO, monomer concentration in reaction solution: (left side in photo) 20 wt%, (right side) 25 wt%, reaction temperature: 45 $^{o}$C, reaction time 48 h.

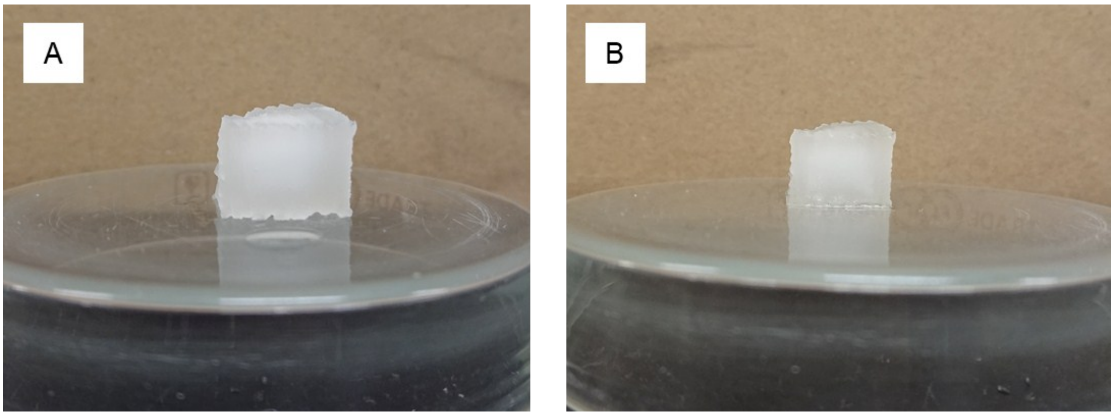


Figure S4 Photos of TPT-HDA porous polymer, (A) dipped in Chloroform, (B) dipped in DMF, [TPT]/[HDA] = 4/3 (mol/mol), solvent of reaction system: DMSO, monomer concentration in reaction solution: 20 wt%, reaction temperature: 45 °C, reaction time 48 h.

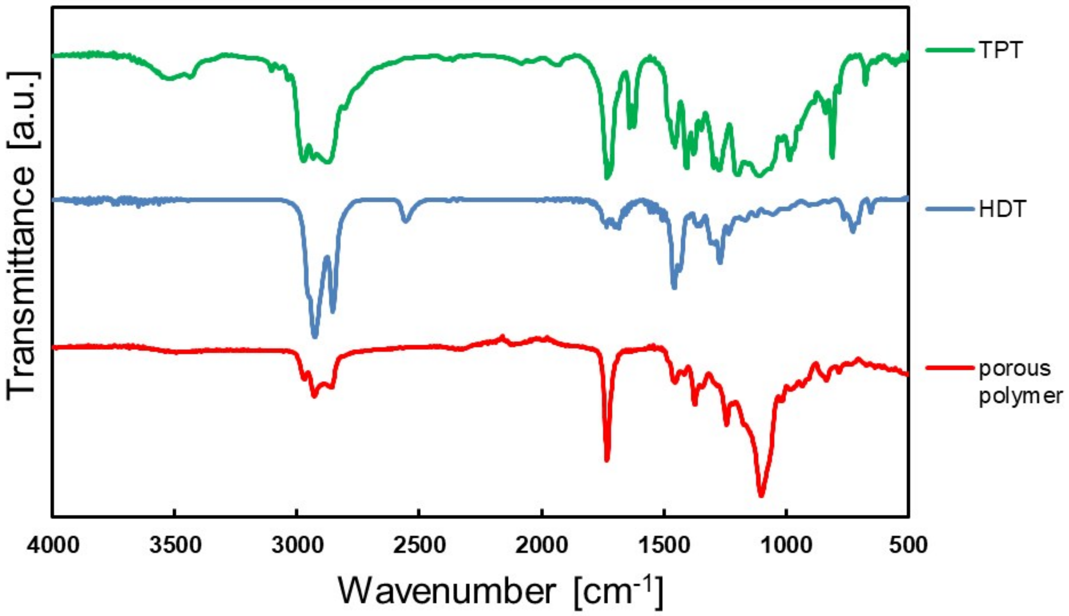


Figure S5 (a) FT-IR spectra of TPT, HDT and TPT-HDT porous polymer, solvent of reaction system: DMSO, monomer concentration in reaction solution: 25 wt%, catalyst concentration in reaction solution: [TEA] = 70.7 µmol/mL, reaction temperature: r.t., reaction time: 48 h.

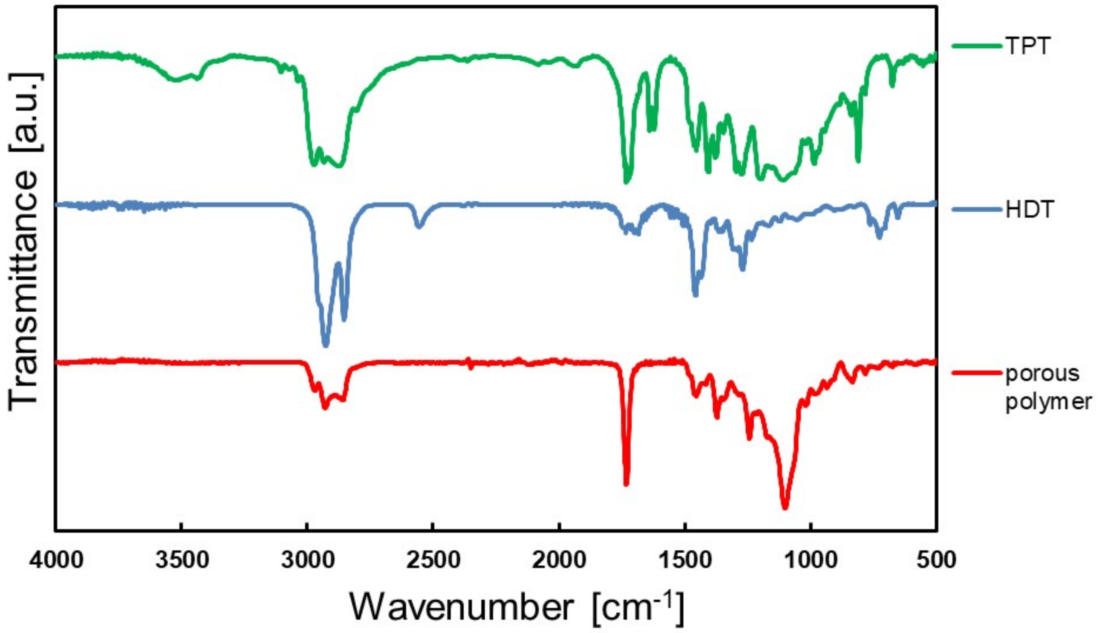


Figure S5 (b) FT-IR spectra of TPT, HDT and porous polymer, solvent of reaction system: DMSO, monomer concentration in reaction solution: 25 wt%, catalyst concentration in reaction solution: [Pip] = 3.33 μmol/mL, reaction temperature: r.t., reaction time: 48 h.

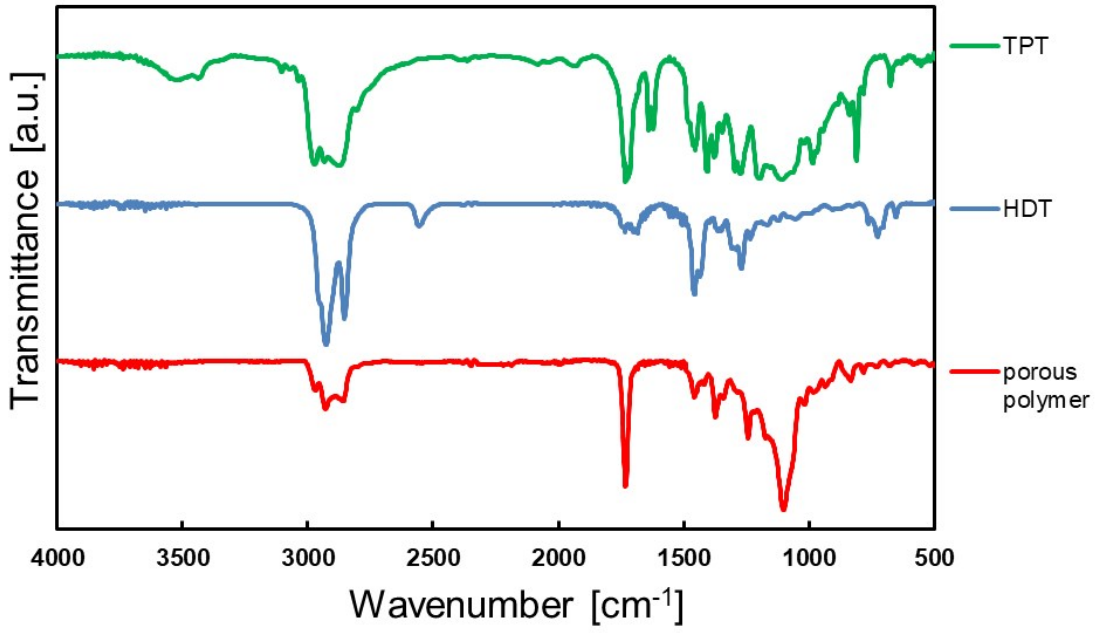


Figure S5 (c) FT-IR spectra of TPT, HDT and porous polymer, solvent of reaction system: DMSO, monomer concentration in reaction solution: 25 wt%, reaction temperature: r.t., catalyst concentration in reaction solution: [WPBG-027] = 1.42 μmol/mL, reaction time: 48 h.

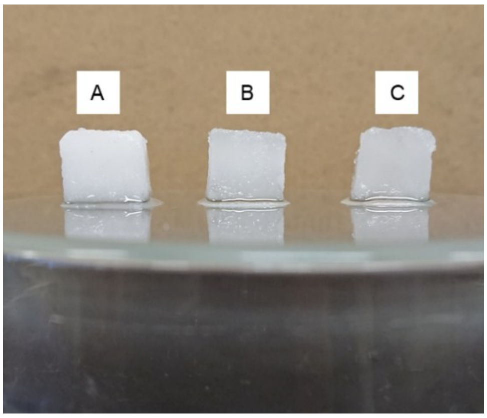


Figure S6  Photos of TPT-HDT porous polymers dipped in DMSO, [TPT]/[HDT] = 2/3 (mol/mol), solvent to prepare: DMSO, monomer concentration in reaction solution: 25 wt%, catalyst concentration in reaction solution: (A) [TEA] = 70.7 μmol/mL, (B) [Pip] = 1.66 μmol/mL, (C) [WPBG-027] = 1.42 μmol/mL, reaction temperature: r.t., reaction time 48 h.
